# Knowledge, attitudes and practices (KAP) towards rabies and free roaming dogs (FRD) in Panchkula district of north India: A cross-sectional study of urban residents

**DOI:** 10.1371/journal.pntd.0007384

**Published:** 2019-04-29

**Authors:** Harish Kumar Tiwari, Ian D. Robertson, Mark O’Dea, Abi Tamim Vanak

**Affiliations:** 1 College of Science, Health, Engineering and Education, School of Veterinary and Life Sciences, Murdoch University, Western Australia, Australia; 2 Ausvet, Fremantle, Western Australia, Australia; 3 Ashoka Trust for Research on Ecology and the Environment (ATREE), Bangalore, India; 4 China-Australia Joint Research and Training Center for Veterinary Epidemiology, Huazhong Agricultural University, Wuhan, Hubei, China; 5 Wellcome Trust/DBT India-Alliance Fellow, Hyderabad, India; 6 School of Life Sciences, University of KwaZulu-Natal, Durban, South Africa; Wistar Institute, UNITED STATES

## Abstract

Canine rabies is endemic in urban India. A questionnaire was administered to 204 residents of the urbanised municipality of Panchkula in north India to assess the influence of gender, age, family size, social status and dog ownership, over the knowledge, attitudes and practices (KAP) towards rabies control and free-roaming dogs (FRD) in their locality. Bivariate analyses revealed significant knowledge gaps regarding crucial information on the control and transmission of rabies. Multivariable logistic regression models found that the respondents with a high/middle socio-economic status were likely to be more knowledgeable than those from low socio-economic levels (OR 3.03, 95%CI 1.5–6.0, p = 0.001). Households with children ≤14 years of age were likely to be lacking in knowledge about rabies compared to households with older or no children (OR 0.5, 95%CI 0.3–0.9, p = 0.04). The attitudes and practices of the respondents towards rabies control was positive in households with a high/middle socio-economic status (OR 3.4, 95%CI 1.7–7.2, p = 0.0008) but poor in older (≥ 35 years) participants (OR 0.4, 95%CI 0.2–0.7, p = 0.001). It is concluded that rabies awareness campaigns should be developed and conducted to target sectors of the urban community such as those belonging to lower socio-economic sections and schools to improve the residents’ knowledge and practices towards rabies. Educating dog owners about sterilising their pets is also recommended to alter the attitudes of the residents towards FRD population control.

## Author summary

An enhanced level of awareness regarding rabies and management of dog-bite wounds is usually expected of urban residents owing to improved health care facilities and superior avenues for information dissemination. This perception can be misleading as gaps were found in the knowledge of residents of the Municipal Corporation Panchkula in north India regarding the disease, especially its modes of transmission and management of dog-bites. Also, there were gaps in the knowledge of the residents regarding the contribution free-roaming dogs make to keeping rabies endemic in the city. Such shortcomings in the knowledge, attitudes and practices towards rabies were prominent in older residents, those from low socio-economic status, and those families who had children ≤ 14 years of age. Dog owners were not aware of the importance of dog population management and responsible dog-ownership. It is recommended that the Municipal Corporation of Panchkula should encourage educational institutions to include material about rabies in the curriculum, and increase the frequency of awareness campaigns to alter the perception of the wider community, including dog owners, towards rabies, its control and dog population management.

## Introduction

An estimated 59,000 human deaths occur annually due to rabies in the world, and 99% of this global mortality is attributed to the transmission of the virus through dog-bites [1]. The disease is endemic in Asia with India reporting the highest number of human deaths within the region, primarily amongst people from rural areas with poor socioeconomic backgrounds [2–4]. However, the true public health impact of rabies in India is unknown due to a lack of accurate data [5]. A gross lack of awareness about the disease is one of the prime factors that leads to under-reporting of human mortality due to rabies [6].

As most rabies prevention centres in India are located in urban areas [7], one would expect lower exposure and higher treatment seeking behaviour against rabies in the urban compared to the rural population. However, rapid urbanisation and inadequate garbage management systems within urban environments facilitates the emergence of rabies through the indiscriminate breeding of free-roaming dogs (FRD) on the city streets [8–10]. These FRD in the urban localities might range from semi-owned dogs that maintain some level of human interaction through the supply of food/shelter, to being completely unrestricted feral dogs that solely depend on scavenging for their existence. The dog population in India grew the fastest in the world during 2007–2012 [11, 12] and the human-FRD conflict in urban areas is evident through the increasing incidence of unprovoked dog-bites [13–18]. Vanak [19], stated that there were fewer cases of rabies in humans in urban India than in rural India due to better availability to post-exposure prophylaxis. However, as rabies is endemic in the FRD population of Indian cities, there remains potential risk of rabies exposure to urban residents through dog-bites [20, 21].

An efficient rabies control programme in urban areas is characterised by application of measures that would involve: mass vaccinations and control of the movements of FRD; control of their reproduction; initiating habitat control measures such as better garbage management; and remove unsupervised dogs [2, 22]. None of these are possible without the active participation of the people whose knowledge, attitudes and practices regarding rabies and FRD may be largely influenced by their religious, cultural or traditional beliefs. The availability and economics of preventive measures, such as anti-rabies vaccine (ARV), rabies immunoglobulins (RIG) and canine vaccines, also influence the uptake of control programmes.

In the wake of the increased FRD population in urban India it is important to assess the KAP of urban communities, not only towards rabies, but also towards FRD, before the disease can be effectively controlled [23]. KAP surveys can help to point out the inadequacies of the existing disease control programmes and help improve their effectiveness by managing the shortcomings. In the current study the KAP of a sample of residents of Panchkula Municipal Corporation in Haryana state, India was undertaken through a cross-sectional survey to assess: (1) the KAP of an urban community towards rabies and its control; (2) the KAP of an urban community towards FRD population management; and (3) the attitudes and practices of an urban dog owning population towards responsible ownership of dogs.

## Materials and methods

### Ethics statement

This study involved survey of residents of Panchkula Municipal Corporation administrated wards (ward 9 to 17) in the state of Haryana, India and the administrative approval of Panchkula Municipal Corporation was obtained for the study while ethical approval was obtained from the Murdoch University Human Ethics Committee (permission number: 20/2016).

### Study area

**Fig 1:**
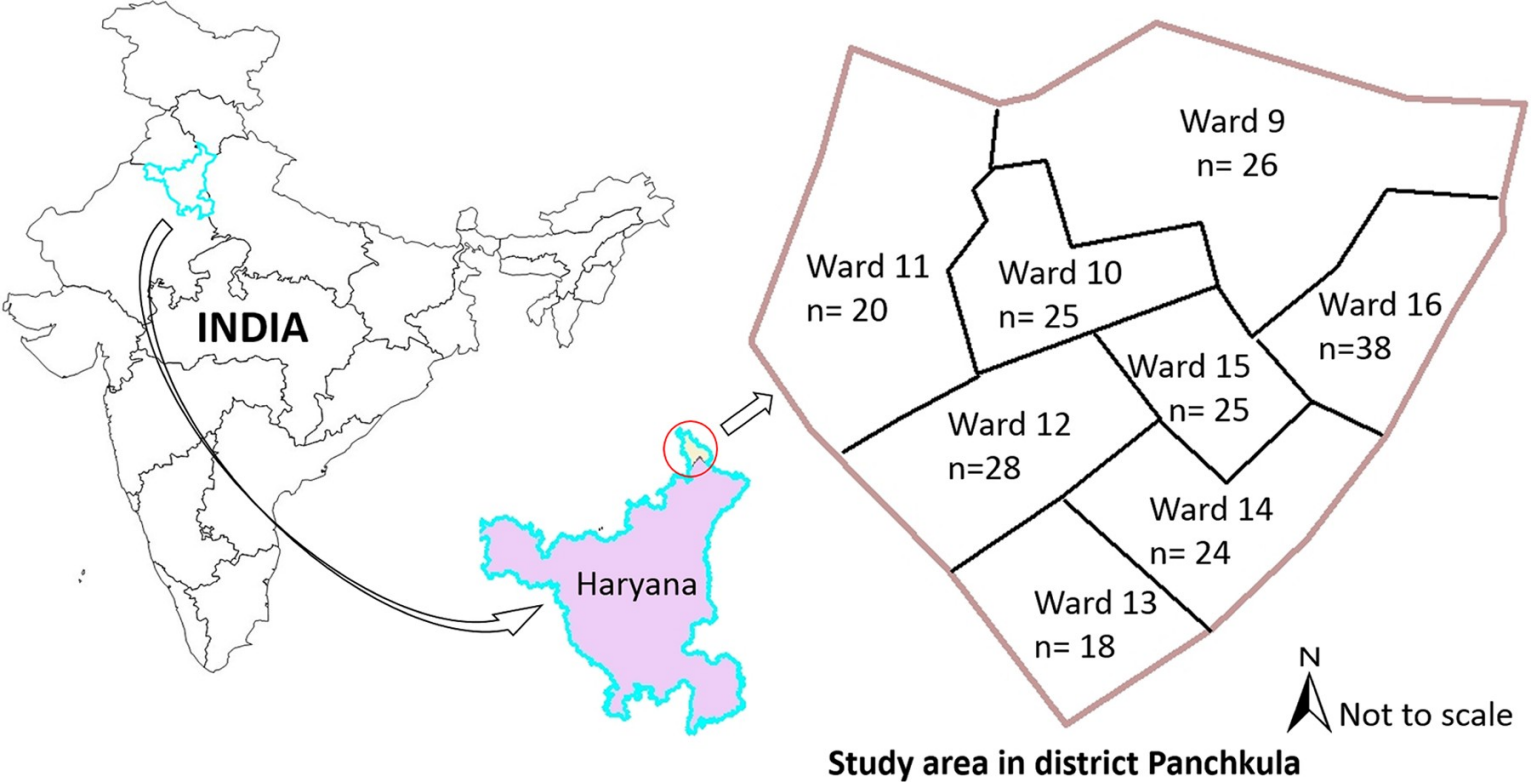
The study area in Panchkula Municipal Corporation, Haryana state, India with the number of households interviewed from each ward (total households interviewed = 204).

A questionnaire was developed and administered to a sample of residents of Panchkula Municipal Corporation, Panchkula district, Haryana state in north India during September-October 2016. In the greater Panchkula district, 54.87% of the population is described as urban, of whom most reside in the wards under the administrative control of Panchkula Municipal Corporation [24]. The total number of households in wards approved for conducting the study was 13,627, with a population of 59,306 people (http://censusindia.gov.in/, as accessed in July 2016). The wards of the Panchkula Municipal Corporation administrated area are numbered 9 to 16 and comprise of highly organised residential, administrative and industrial habitats interspersed with unorganised slums and villages [25]. Ward 11 comprises of slums and urban villages with non-numbered houses, while the houses in the other wards are numbered. The number of households in the wards varies from 1,016 (ward 13) to 2,322 (ward 16) (Fig 1). Administrative approval for conducting the study in the eight wards was obtained from the Panchkula Municipal Corporation.

### Sample size

The target sample size for this study was based on the weighted average of three previous KAP surveys conducted in urban India by calculating the average number of participants with awareness of rabies from these studies [26–28]. 87.8% of the households were assumed to be aware of rabies, and for a 95% confidence and 5% error rate, a sample size of 172 was determined from the 13,627 present in Panchkula (http://epitools.ausvet.com.au, accessed 23 April 2016). We approached slightly more households (204) to cater for refusals (2) or partially completed surveys (7).

### Sampling procedure

The number of households selected from each ward was in direct proportion to the total number of households in that ward and ranged from 18 to 38. The households were randomly selected from sectors of each ward using a random number generator, except for ward 11 where the houses were not numbered, and from where 20 households were selected through a rolling sampling method [29].

The head of the household was invited to participate; however if the household head was not present, the oldest adult member of the family/household (> 18 years of age) was surveyed in a face-to-face situation. Prior to the commencement of the survey, the study was explained to the participants, the confidentiality of their answers confirmed and oral consent to participate obtained.

### Questionnaire design

The aim of the KAP questionnaire was to: identify gaps in the knowledge about rabies; assess the practices of the urban residents towards the disease that potentially contributes to the persistence of rabies; evaluate the attitudes of the respondents towards FRD; and assess the attitudes and practices of urban dog owners towards their pets. The questionnaire consisted of closed questions on: (a) the demographic characteristics of the household; (b) KAP regarding rabies (16 questions of which 11 pertained to knowledge and five to attitudes and practices towards rabies, respectively); (c) attitudes and practices towards FRD (seven questions); and (d) pet care practices adopted by the dog owners (15 questions). The questions were read out to the respondents in their local language (Hindi) by the interviewer and their answers were recorded in English (Appendix). The questionnaire was approved by the Murdoch University Human Ethics Committee (2016/20).

### Data management and analysis

The responses on the questionnaire sheet were transferred onto an EXCEL spreadsheet (Microsoft Excel, Microsoft Corp., Redmond, WA, USA) and made compatible for subsequent analysis using the software R [30]. A matrix was developed to categorise the respondents into high, middle, and low socio-economic status on the basis of their educational qualification and occupation on a design based on www.praja.org (accessed 18 March 2016). Subsequently, the high and middle categories were merged to obtain a binomial distribution of respondents into two socio-economic divisions: low and high/middle (S1 Table). The age of the respondents and the family size of the households were each dichotomised into two groups based on the median age/family size.

A bivariate analysis of the responses of the participants to the individual questions pertaining to knowledge of rabies, attitudes and practices regarding rabies control and attitudes towards FRD was carried out using a χ^2^ or Fisher’s exact test. The residents were categorised as having adequate or inadequate knowledge of rabies; positive or negative attitudes and practices towards controlling rabies; and positive or negative attitudes towards FRD based on the median score to the responses to the questions pertaining to the relevant sections of the questionnaire. The associations between this outcome and the various descriptive variables were initially evaluated with the χ^2^ or Fisher’s exact test. All descriptive variables with a p ≤ 0.25 were then offered to multivariable logistic regression models. Reduced subset models were developed using backward elimination based on the AIC (Akaike Information Criteria) score. The final multivariable logistic regression models were evaluated using Pearson’s and Deviance residuals and goodness-of-fit was assessed by the Hosmer-Lemeshow test [31].

## Results

A total of 204 respondents completed the questionnaire and their descriptive characteristics are summarised in Table 1.

**Table 1:** Demographic characteristics of respondents in Panchkula, India, 2016.

Variable/Category	n (%)
**Gender**	
Male	120 (59)
Female	84 (41)
**Age (years)**	
18–34	73 (36)
≥ 35	131 (64)
**Socio-economic status**	
High/middle	153 (75)
Low	51 (25)
**Family size**	
≤5	147 (70)
≥6	57 (30)
**Family contains children ≤ 14 years**	
Yes	121 (59)
No	83 (41)
**Dog ownership**	
Yes	74 (36)
No	130 (64)

The median score for correct responses towards knowledge of rabies; attitudes and practices towards rabies control; and attitudes towards FRD were 8, 3 and 3 respectively.

The univariable analyses (χ^2^ test) of responses pertaining to knowledge of rabies that was asked of 195 (96%) respondents are displayed in Table 2. The variables gender (p = 0.2), socio-economic status (p = 0.0003), families with and without children ≤ 14 years of age (p = 0.01) and dog ownership (p = 0.14) were offered to the multivariable logistic regression model to assess the participant’s knowledge (Table 3). The model was shown to adequately fit the data with a Likelihood ratio (χ^2^) test of 17.6 (p = 0.00015) and a Hosmer–Lemeshow goodness of fit test value of 0.01 (p = 0.91).

**Table 2:** Test of association (χ^2^) between knowledge about rabies and various predictor variables in Panchkula, India, 2016.

Variable/category	N (%)	Number knowledgeable n (%)	P-value	OR (95% CI)
Gender				
Female	84 (41)	51 (61)		1.0
Male	120 (59)	62 (52)	0.2*	0.7 (0.4–1.2)
Age (years)				
≤ 34	73 (36)	38 (52)		1.0
≥35	131 (64)	75 (57)	0.47	1.2 (0.7–2.2)
Socio-economic status				
Low	51 (25)	17 (33)		1.0
High/middle	153 (75)	96 (63)	0.0003*	3.3 (1.7–6.6)
Family size				
≤5	147 (70)	82 (56)		1.0
≥6	57 (30)	31 (54)	0.86	1.05 (0.6–2.0)
Children ≤ 14 years				
No	83 (41)	55 (66)		1.0
Yes	121 (59)	58 (48)	0.01*	0.5 (0.3–0.8)
Dog ownership				
No	130 (64)	67 (51)		1.0
Yes	74 (46)	46 (62)	0.14*	1.5 (0.9–2.8)

N = total respondents, n = respondents having a knowledge score of ≥8,

* Variables offered to the initial saturated model.

**Table 3:** Multivariable logistic regression model of factors associated with the participants’ knowledge of rabies in Panchkula, India, 2016.

Variable/category	Coefficient (b)	Standard Error	P-value	OR (95% CI)
Constant	-0.24			
**Socio-economic status**				
Low				1.0
High/middle	1.11	0.34	0.001	3.03 (1.5–6.0)
**Households with Children ≤ 14 years**				
No				1.0
Yes	-0.6	0.3	0.04	0.5 (0.3–0.9)

Likelihood ratio (χ^2^) test = 17.6; p = 0.0001; Hosmer–Lemeshow goodness of fit test = 0.01 (p = 0.91)

In the bivariate analyses of responses about knowledge on rabies (S2 Table), respondents from the low socio-economic group were less likely to: have heard of rabies (OR 0.1 95%CI 0.02–0.4, p = 0.001); and know that the disease was fatal (OR 0.5, 95%CI 0.2–0.9, p = 0.02) or preventable (OR 0.2 95%CI 0.1–0.5, p = 0.001). They also were less knowledgeable about: the role dogs played in rabies transmission (OR 0.15, 95%CI 0.04–0.6, p = 0.008); the importance of administering human prophylaxis, such as PEP (OR 0.2, 95%CI 0.1–0.5, p = 0.001); and the control of rabies through vaccination of dogs (OR 0.3, 95%CI 0.1–0.5, p = 0.001). Younger respondents (18–34 years of age) were more likely to know of the possible transmission through licks/scratches from infected animals (OR 1.9, 95%CI 1.1–3.5, p = 0.02). Households with children ≤ 14 years of age were less aware of rabies transmission through animal bites or through licks or scratches from infected animals (OR 0.27, 95%CI 0.05–0.9, p = 0.04 and OR 0.57, 95%CI 0.3–1.0, p = 0.05, respectively). Dog-owners were more aware of the usefulness of vaccination of dogs to prevent rabies (OR = 3.0, 95%CI 1.4–7.1, p = 0.003) than non-dog owners.

The variables age of the respondents (p = 0.003), socio-economic status (p = 0.001), family size (p = 0.24) and households having children ≤ 14 years of age (p = 0.06) were offered to the multivariable logistic regression to assess participant’s attitudes and practices towards rabies control based on the univariable analysis (Table 4). The model was shown to be a good fit of the data (Hosmer-Lemeshow goodness of fit test = 0.04; p = 0.83) (Table 5).

**Table 4:** Test of association (χ^2^) between the respondents’ attitudes and practices towards better control and prevention of rabies and their attitudes towards free-roaming dogs, and various predictor variables in Panchkula, India, 2016.

Variable/category	N (%)	Respondents with positive attitudes and practices towards rabies, n (%)	P-value	OR (95% CI)	Respondents with positive attitudes towards free-roaming dogs, n (%)	P-value	OR (95% CI)
Gender							
Female	84 (41)	41 (49)		1.0	14 (17)		1.0
Male	120 (59)	51 (42.5)	0.37	0.8 (0.4–1.3)	19 (16)	0.87	0.9 (0.4–2.0)
Age (years)							
≤ 34	73 (36)	43 (59)		1.0	9 (12)		1.0
≥ 35	131 (64)	49 (37)	0.003*	0.4 (0.2–0.7)	24 (18)	0.26	1.5 (0.7–3.8)
Socio-economic status							
Low	51 (25)	13 (25)		1.0	9 (18)		1.0
High/middle	153 (75)	79 (52)	0.001*	3.08 (1.5–6.4)	24 (16)	0.74	0.8 (0.4–2.1)
Family size							
≥6	147 (70)	70 (48)		1.0	22 (15)		1.0
≤ 5	57 (30)	22 (39)	0.24*	1.4 (0.8–2.7)	11 (19)	0.45	0.73 (0.3–1.7)
Children ≤ 14 years							
No	83 (41)	44 (53)		1.0	11 (13)		1.0
Yes	121 (60)	48 (40)	0.06*	0.6 (0.3–1.0)	22 (18)	0.34	1.4 (0.7–3.3)
Dog ownership							
No	130 (64)	60 (46)		1.0	23 (18)		1.0
Yes	74 (46)	32 (43)	0.69	0.9 (0.5–1.6)	10 (13)	0.43	0.7 (0.3–1.6)

N = total respondents, n = respondents scoring knowledge score of >3,

* Variables offered to the initial saturated model.

**Table 5:** Multivariable logistic regression model of factors associated with the respondents’ attitudes and practices towards rabies control and prevention in Panchkula, India, 2016.

Variable/category	Intercept(b)	Standard Error	P-value	OR(95% CI)
Constant	-0.53			
Age (years)				
≤ 34				1.0
≥35	-0.98	0.31	0.001	0.4 (0.2–0.7)
Socio-economic status				
Low				1.0
High/middle	1.24	0.37	0.0008	3.4 (1.7–7.2)

Likelihood ratio (χ^2^) test = 21.2; p<0.0001; Hosmer–Lemeshow goodness of fit test = 0.04; p = 0.83

The bivariate analyses of the responses regarding attitudes and practices of the respondents towards rabies that help its control are presented in S3 Table. Younger respondents (≤ 34 years) were more likely to use soap and water to wash dog-bite wounds (OR 2.7, 95%CI 1.4–5.1, p = 0.001) and inform the municipal authorities if they came across a dog displaying rabies-like signs (OR 2.7, 95%CI 2.7–5.0, p = 0.001) than older respondents (≥ 35 years). Respondents from the high/middle socio-economic class were more likely to: approach a hospital in the event of a dog bite (OR 3.8, 95%CI 1.7–8.6, p = 0.01); inform municipal authorities about sighting a dog showing rabies-like-clinical signs (OR 3.8, 95%CI 1.9–7.4, p = 0.001); and believe that restricting the population of FRD would help control rabies (OR 3.8, 95%CI 1.9–7.5, p = 0.001) than those from a low socio-economic class.

In the univariable analysis of the attitudes and practices towards FRD, none of the explanatory variables, when tested for association with the response variable, had p-values < 0.25 (Table 4), hence developing a multivariable logistic regression model was not attempted.

The bivariate analyses of responses regarding attitudes and practices of the respondents towards FRD are summarized in S3 Table. Dog-owners (OR 2.3, 95% CI 1.2–4.4, p = 0.008) and older residents (≥ 35 years) (OR 2.4, 95%CI 1.3–4.5, p = 0.006) believed that FRD were useful to society while younger respondents (≤ 34 years) (OR 2.01, 95%CI 1.1–3.7, p = 0.02), households containing children ≤ 14 years (OR 1.9, 95%CI 1.06–3.5, p = 0.03), and households with a low socio-economic level (OR 2.7, 95%CI 1.3–6.4, p = 0.01) considered that FRD were a problem to their society. Younger respondents (≤ 34 years) were less likely to take an injured dog to a veterinarian (OR 0.3, 95%CI 0.2–0.6, p = 0.002) than older respondents. More male (OR 2.2, 95%CI 1.1–4.4, p = 0.02) than female respondents considered FRD a threat to human health. In contrast, respondents from the high/middle socio-economic group (OR 0.3, 95%CI 0.1–0.9, p = 0.02) and dog owners (OR 0.5, 95%CI 0.2–0.9, p = 0.03) did not feel that FRD were a threat to human health. Details of the attitudes and practices of the urban residents towards FRD are presented in Table 6. A significant association was found between respondents who have good knowledge and those who have positive attitudes about rabies control (OR 3.2, 95% CI 1.8–5.8, p<0.001).

**Table 6:** Respondents’ responses to various questions pertaining to attitudes and practices relevant to free roaming dogs in Panchkula, India, 2016.

Criteria		n (%)
Are there FRD in your locality?		204 (100)
**Source of FRD **		
Breeding of local FRD		115 (56)
Nearby villages		74 (36)
Pets abandoned by villagers		15 (8)
**FRD are useful for the society**		55 (27)
For guarding premises*		46 (84)
Keep away wild animals*		5 (9)
Keep away thieves*		14 (25)
FRD are a nuisance to the society		138 (68)
FRD are neither useful nor a problem		11(5)
FRD are a threat to human health		160 (78)
Food source for FRD*		
Garbage dumps		106 (52)
Edible street litter		43 (21)
Fed by residents		109 (53)
**Respondents who would feed a FRD**		148 (72)
Reasons why respondents would feed a FRD*		
Religious reasons		57 (37)
Compassion		118 (80)
Better than wasting the left-over food		114 (77)
**Condition of the FRD# **		
Good		39 (19)
Average		108 (53)
Poor		57 (28)
Would take an injured FRD to a veterinarian		78 (38)
Residents who feed/shelter FRD should be responsible for their health/vaccination	Yes	111 (54)
	No	93 (46)
Health/vaccination of the FRD is the responsibility of the government?	Yes	182 (89)
	No	22 (11)
Best way to control FRD population*		
Culling		8 (4)
Impounding		53 (26)
Animal Birth Control		139 (68)
Garbage management		50 (24)

*Respondents could choose more than one option

Seventy-four (36%) of the survey participants owned one or more dogs (total of 91 dogs owned). Most (67) owned one dog, three residents owned two, three residents owned four, and one resident owned 6 dogs. The details of the owned dogs are presented in Table 7. Respondents from the low socio-economic status were less likely to own a dog (OR 0.3, 95%CI 0.1–0.7, p = 0.004) and if they did it was less likely to be a pedigree dog (OR 0.2, 95%CI 0.07–0.5, p = 0.008).

**Table 7:** The characteristics of owned pet dogs and the owner's perceptions and practices about their pets in Panchkula, India, 2016.

Criteria	Number (%)
**Gender of the dog(s) owned** **^**	
Male	75 (82)
Female	16 (18)
**Breeds of dog(s) owned** **^**	
Pedigreed	62* (68)
Local	22 (24)
Mixed breed	7# (8)
**Source of owned dogs** **^**	
Purchased	48 (53)
Gifted	12 (13)
Adopted	26 (29)
Offspring of owned pet	5 (5)
**Respondents who preferred pedigree dog to local breed**	60 (81)
**Reasons for preferring a pedigree dog**	
Intelligence~	24 (40)
Cleanliness~	12 (20)
Social status~	11 (18)
Combination of above reasons	13 (22)
**Are the dog(s) registered** **^**	
Yes	52@ (70)
No	39 (30)
**Are the dog(s) confined and restricted?** **$**	
Yes	65 (88)
No	9 (12)
**Are the dog(s) supervised when not confined/restricted?** **$**	
Always	49 (66)
Sometimes	15 (20)
Rarely	10 (14)
**Respondents who visited a veterinarian in the last year**	67 (90)
**Number of dogs vaccinated against rabies** **^**	65 (71)
**Number of households with sterilised dogs** **$**	7 (8)
**Respondents’ reasons for not sterilising an owned dog** **$**	
Unaware of the procedure	1 (2)
Unavailability of the service	2 (3)
Consider it a cruel practice	8 (11)
Pet reared for breeding	14 (19)
Cost of the procedure	8 (11)
Pet too young for the procedure	2 (3)
No specific reason	32 (43)

*19 Labradors, 15 German Shepherd dogs, 9 Pugs, 7 Pomeranians, 3 each of Rottweilers and Cocker Spaniels and one each of Bull Terrier, Dobermann Pinscher, German Spitz, Irish setter, Chihuahua and Himalayan Gaddi dog;

# cross between pedigreed and FRD;

**~** Respondents chose more than one option;

**@** Registered with Kennel Club of India;

^Total number of owned dogs = 91;

$ Total number of dog-owning respondents = 74

## Discussion

We identified a number of factors of interest regarding the KAP of respondents in Panchkula, in particular: (a) the rabies awareness level of households from the low socio-economic level and those with children ≤14 years is significantly low; (b) the respondents in the higher age group (≥35 years) and households from the low socio-economic level have gaps in the attitudes and practices towards rabies control; and (c) dog-owning residents prefer a pedigree dog than a FRD, however, they would provide food and shelter to FRD due to compassion for them.

### Community knowledge and awareness of rabies

A high proportion (96%) of respondents had heard of rabies, an increase from that reported in urban localities in India (69 and 74%) by Ichhpujani, Chhabra [32] and Herbert, Basha [28]. More recent studies, report a higher proportion (84%), albeit lower to the present study [27, 33]. Elsewhere, international studies have reported comparable levels of awareness viz. Sri Lanka (90%); Bali, Indonesia (97%); and 94% in the Bohol Province, Philippines [34–36]. This rise has been largely attributed to wide dispersion of information via television and radio sources [36]. While these reasons apply to increased cognisance in India as well, it may also be linked to the Government of India prioritizing rabies as a disease of importance and its inclusion in the Nation’s recent five year plan [7]. In Panchkula, information on rabies is spread by the Municipal Corporation through awareness campaigns, rallies and awareness quizzes in schools (personal communication, Municipal Commissioner, Panchkula). Although these measures are intended to increase the awareness about rabies, they are also instrumental in more residents taking notice of the disease, even when their knowledge about various aspects of the disease remains incomplete.

In spite of Panchkula being one of the well planned and organised municipal towns in India [24], with a literacy rate higher than the national average (http://www.schooleducationharyana.gov.in/, accessed 30 June 2017); the respondents lacked understanding of the disease, especially regarding its mode of transmission, methods to prevent infection after dog-bites, and the possibility of disease transmission by animals other than dogs or by licks and scratches from a rabid animal (S1 Table). Such knowledge gaps not only contradicts the presumption that urban dwellers are well informed about the disease but also reflects the inconsistent reach of the awareness programmes in different sections of the population. Similar findings have also been reported in Dehradun (23.7%) and Delhi (42%) [27, 37]. The respondents in Panchkula were, however, better informed about the prophylactic rabies immunisation for dogs and PEP for humans compared to studies from elsewhere in the country [26, 27, 37]. Nonetheless, a lack of vital information on transmission of rabies virus in the residents may also be due to the prime focus of measures initiated by the Municipal authorities towards control of the FRD population rather than concerted efforts to enhance general awareness of the community about rabies as a disease (personal communication, Executive officer, Panchkula Municipal Corporation). A shift in focus is recommended to make the residents aware of the routes of transmission and also simple measures that can possibly prevent rabies, such as washing of dog-bite wounds with soap and water. Awareness of the disease and processes to adopt can be improved by increasing the visibility of information through posters, print and mass media as reported in villages near Bangalore in southern India following use of Information, Education and Communication (IEC) material to enhance knowledge about rabies [38]. Introduction of rabies information sessions in schools also helps improve knowledge and awareness about the disease as has been demonstrated in Sikkim, India [39]. As the present study reinforced the role of the socio-economic status on a participant’s knowledge (Table 2), awareness campaigns need adjusting to target disadvantaged groups [28, 40]. A smaller number of respondents (16) recalled the running of awareness campaigns on rabies, of which only two were from low socio-economic sections of the society implying that the awareness campaigns are neither far-reaching, nor targeting the low socio-economic sector.

Conversely, there are some positive outcomes from this study, such as no significant difference between the knowledge about rabies in males and females in Panchkula (S1 Table). This may be due to equal opportunity of males and females to acquire information on rabies, as opposed to the better opportunities offered to males to gather knowledge regarding rabies as reported in some studies such as in Ethiopia [41, 42]. The prospect of equality of knowledge dissemination among genders in Panchkula could be capitalised to spread information amongst women with children as it was found that families with children of vulnerable ages (≤14 years) lacked adequate understanding of rabies. As the currently employed tool for spreading awareness in Panchkula Municipality is primarily mass media, which has similar exposure opportunities to all groups in the community, irrespective of gender or age, a targeted approach to enhance knowledge, such as in educational institutions, is recommended to educate school children about the methods of rabies virus transmission [39].

Dog ownership was found to be a trait of the economically well off members of the urban society. The likelihood of a dog-owner belonging to a low socio-economic level in Panchkula was found to be low (OR 0.34, p = 0.04), and this explains why dog ownership is not an influencing factor for having a high knowledge score in the multivariable model.

### Community attitudes and practices towards rabies

Failure to wash dog-bite wounds with soap and water by a large proportion of the respondents (39%) reinforces the observation by others [43, 44] that a large section of society is unaware of a simple procedure that can help reduce the incidence of rabies substantially. It is not surprising that more than half of the respondents (113, 55.4%) favoured the use of traditional healing applications, such as chilli powder and turmeric, similar to studies reported elsewhere in urban India including Delhi (51%) [45] and Dehradun (57%) [27]. It is important that awareness campaigns should emphasise that, although turmeric may have antiseptic properties [46], it is not able to kill the rabies virus, which has higher chances of being destroyed if wounds are properly washed with soap and water.

The urban respondents favoured restricting the FRD population but lacked genuine concern for controlling rabies as demonstrated by their negative perception to questions pertaining to practices regarding the disease (S3 Table). In contrast, a KAP study in Bhutan by Dhand, Rai (29) found that most respondents who favoured FRD population control (99.7%) also reported cases of canine rabies to the authorities (98.8%) and practiced washing dog-bite wounds with soap and water (85.4%). In another recent study in Bhutan, the public demand for formulating legislation that could control the FRD population also implied a high level of awareness and a responsible attitude towards controlling rabies [47, page 99], which unfortunately was lacking in Panchkula. A positive aspect of the respondents in the present study, however, was their PEP seeking behaviour (85%), which was similar to a survey in Eluru district (85.5%) in Andhra Pradesh and far higher than the findings of participants from Delhi slums (27.6% and 26.5%), Pune (24%), and Dehradun (55%) [26, 27, 37, 45, 48]. This response is largely driven by easy accessibility to hospitals/clinics in the vicinity of Panchkula, compared to the previously mentioned areas that do not have close access to hospitals.

The socio-economically better off respondents were more likely to seek hospital treatment (OR 3.83, p<0.001), alert the municipal authorities of the presence of a rabid dog (OR 3.79, p<0.001) and support measures restricting the stray dog population (OR 2.49, p = 0.01, S3 Table). This is not unexpected, as better knowledge about the disease should translate into adoption of better practices, as we found a significant association between respondents with good knowledge about rabies and those with positive attitudes about rabies control (OR 3.21, p<0.001).

The older respondents (≥ 35 years of age) were found to have inadequate knowledge regarding the measures that can control/prevent rabies (OR = 0.37, p = 0.001). The low literacy level in the older generation and limited access to information about health and diseases via the internet may be reasons for this lack of knowledge and positive attitudes. This, once again, warrants targeting older people by formulating a structured and sustained information campaign in urban centres, if the knowledge and practices of older residents are to be improved to reduce the incidence of rabies throughout the country.

### Community attitudes and practices towards free roaming dogs

The majority of the respondents (68%) in Panchkula considered FRD a problem, which was consistent with recent findings in Bhutan [47, 70%] but was lower than that reported in Abruzzo, Italy (90%) by Slater, Di Nardo [49]. This difference is most likely because of the low socio-economic standing of participants included in the current study. However, in this study, over a quarter of the respondents felt that FRD were useful (27%) which may be due to the higher sense of security that the urban residents apparently desire, as guarding of the house premises was the most quoted utility (83.6%) by the respondents for the usefulness of FRD.

A prominent finding in this study was that 72.5% of the respondents admitted to feeding FRD, with most (80%) doing this out of compassion, probably linked with the perceived poor welfare of these dogs as only 19% of the respondents felt that FRD were of good health. We feel that factors, other than those included in this study, influence the attitudes of the residents towards FRD, as none of the predictors included in this study were significant. The spatial distribution of the FRD was concentrated in the vicinity of community shopping centres and it is likely that the distance of the households from the shopping centres could be a significant factor that may influence the residents’ attitudes. The city has seen many rabies awareness campaigns and dog population control interventions over the past decade, however, the programmes are often interrupted owing to failure of contracts or insufficient personnel or other resources (personal communication, Executive officer, Panchkula Municipal Corporation).

### Characteristics of urban dog owners

Most dog owners (68%) kept pedigree dogs and not surprisingly, more owned dogs were purchased (53%) than were adopted (29%) off the streets. The rise in dog ownership in India has resulted from the increased income of urban Indian residents [12]. The current study found that dog owners preferred to register their dogs with the Kennel Club India (KCI) but not their local municipality implying that registration is considered important for commercial purposes and eligibility for entering in dog shows rather than enforcing responsible dog ownership. As most dogs were pedigree and purchased, it was expected that the number of dog owners supervising their dogs when they were not restricted/confined would be high (66%). Although a majority (71%) of owned dogs were vaccinated against rabies, only 8% of them were sterilised and many dog owners (43%) could not cite any specific reasons for not having their dog sterilised. This highlights the need for spreading information on dog population control, as well as rabies, in Panchkula.

This KAP survey in Panchkula highlights that contrary to the expected belief that urban residents are better informed about rabies, significant gaps persist in their knowledge towards the disease, especially regarding the means of transmission through licks and scratches of rabid animals in residents of low socio-economic level and in families with children of vulnerable age (≤ 14 years). Inadequate practices regarding rabies prevention were found in the older urban respondents (≥35 years of age) and those from the low socio-economic status. We recommend that in addition to the holistic efforts to spread awareness about rabies, a targeted focus on the sections of the society such as slum residents, primary schools and unskilled workers of industrial sectors should be adopted by the Municipal Corporation to improve the knowledge of these community sectors. Implementing compulsory registration of pet dogs by Municipal Corporation will help in the monitoring of vaccination coverage of owned dogs. An incentive of free vaccination against rabies could also be started by the Municipal Corporation to reduce infection in dogs and hence the human community. Apart from wider participation of educational institutes, it is suggested that the frequency of awareness campaigns should be also increased to alter the perception of the wider community, including dog owners, towards rabies and its control and management of the dog population.

This KAP survey, however, had some short-comings. We suspect that some of the responses may not reflect the true picture, as people sometimes do not practice what they say, e.g. we feel a higher percentage of residents feed a FRD but did not acknowledge this due to the prevailing feeling by the community of their nuisance in this locality. Many dog-owners from high/middle socio-economic sections usually asked the household servants to answer the questionnaire on the pretext that in most cases it is the servant who takes care of their dog. This potentially introduced a bias as the servants do not necessarily represent the awareness and knowledge level of the dog owners. Also, the questionnaire did not explore the reasons for the 29% of dog owners failing to have their pets vaccinated against rabies which is an important aspect that should be included in future surveys. It is recommended that follow-up surveys are conducted including sampling more people per household to confirm the perceptions of urban residents towards FRD. Also we accept that comparisons made in this study with other surveys must be interpreted with caution as the questionnaires and statistical methods vary between studies. Enlarging the sample size and repeating the surveys in other urban areas may overcome such limitations for future KAP surveys.

## Supporting information

S1 Table The matrix developed to categorise the respondents into high, middle and low socio-economic groups. (DOCX)

S2 Table Descriptive and bivariate analyses (χ^2^) of the response to the individual questions relating to knowledge about rabies. (DOCX)

S3 Table Descriptive and bivariate analyses (χ^2^) of the response to the individual questions relating to attitudes and practices about rabies. (DOCX)

S4 Table Descriptive and bivariate analyses (χ2) of the response to the individual questions relating to attitudes and practices towards free-roaming dogs. (DOCX)
