# Supplementary material for: Knowledge, attitudes and practices (KAP) towards rabies and free roaming dogs (FRD) in Panchkula district of north India: A cross-sectional study of urban residents
Source: PLoS Negl Trop Dis. 2019 Apr 29;13(4):e0007384. doi: 10.1371/journal.pntd.0007384 (PMC6508743; doi:10.1371/journal.pntd.0007384)
Supplement: S1 Table — (DOCX) [file pntd.0007384.s001.docx]

Table S1. The matrix developed to categorise the respondents into High, middle and low socio-economic groups ([www.praja.org](http://www.praja.org))

| Education level Occupation/Trade | No Formal  Education | PRIMARY | SECONDARY | MATRICULATE | COLLEGE | GRADUATE | POST -GRADUATE |
| --- | --- | --- | --- | --- | --- | --- | --- |
| UNSKILLED | F | F | F | E | E | E | D |
| SKILLED | F | F | E | E | E | D | D |
| SMALL TRADING | F | E | E | E | D | D | D |
| SHOP OWNER | E | E | E | D | D | D | C |
| BUSINESS | E | E | D | D | D | C | C |
| CLERICAL | E | D | D | D | C | C | C |
| SUPERVISOR | D | D | D | C | C | C | B |
| OFFICER/EXECUTIVE | D | D | C | C | C | B | B |
| SR.OFFICER | D | C | C | C | B | B | B |
| PROFESSIONAL | C | C | C | B | B | B | A |

A/B – High socio-economic group; C/D – Middle socio-economic group; E/F – Low socio-economic group
