# Supplementary material for: Knowledge, attitudes and practices (KAP) towards rabies and free roaming dogs (FRD) in Panchkula district of north India: A cross-sectional study of urban residents
Source: PLoS Negl Trop Dis. 2019 Apr 29;13(4):e0007384. doi: 10.1371/journal.pntd.0007384 (PMC6508743; doi:10.1371/journal.pntd.0007384)
Supplement: S2 Table — (DOCX) [file pntd.0007384.s002.docx]

| **Variable** |  | **Gender** | | **P-value** | **Age of respondents** | | **P-value** |
| --- | --- | --- | --- | --- | --- | --- | --- |
|  | **N= 204** | **Male** | **Female** |  | **≤34**  **years** | **≥35 years** |  |
| Have you heard about rabies? | |  |  | **0.49** |  |  | **0.28** |
| Yes | 195 | 116 | 79 |  | 127 | 68 |  |
| No | 9 | 4 | 5 |  | 4 | 5 |  |
| Can dogs transmit rabies? |  |  |  | **0.74** |  |  | **0.28** |
| Yes | 195 | 114 | 80 |  | 127 | 68 |  |
| No | 9 | 6 | 3 |  | 4 | 5 |  |
| Can cats transmit rabies? |  |  |  | **0.45** |  |  | **0.82** |
| Yes | 67 | 37 | 30 |  | 41 | 26 |  |
| No | 137 | 83 | 54 |  | 90 | 47 |  |
| Can rats transmit rabies? |  |  |  | **0.62** |  |  | **0.35** |
| Yes | 67 | 38 | 29 |  | 46 | 21 |  |
| No | 137 | 82 | 54 |  | 85 | 52 |  |
| Can rabies be transmitted through animal bites? | | |  | **0.61** |  |  | **0.5** |
| Yes | 186 | 108 | 78 |  | 117 | 69 |  |
| No | 18 | 12 | 6 |  | 14 | 4 |  |
| Can rabies be transmitted through licks/scratches? | | |  | **0.99** |  |  | ***0.02**** |
| Yes | 94 | 55 | 38 |  | 68 | 26 |  |
| No | 110 | 65 | 45 |  | 63 | 47 |  |
| Is rabies fatal? |  |  |  | **0.7** |  |  | **0.22** |
| Yes | 142 | 80 | 62 |  | 95 | 47 |  |
| No | 62 | 40 | 22 |  | 36 | 26 |  |
| Can rabies be prevented? |  |  |  | **0.86** |  |  | **0.76** |
| Yes | 162 | 94 | 68 |  | 102 | 60 |  |
| No | 42 | 26 | 16 |  | 29 | 13 |  |
| Can rabies be prevented by post -bite anti-rabies vaccines (ARV)? | | |  | **0.87** |  |  | **0.61** |
| Yes | 155 | 90 | 65 |  | 101 | 54 |  |
| No | 49 | 30 | 19 |  | 30 | 19 |  |
| Can rabies be prevented by vaccinating dogs against rabies? | | |  |  |  |  |  |
| Yes | 156 | 93 | 63 | **0.67** | 99 | 57 | **0.68** |
| No | 48 | 27 | 21 |  | 32 | 16 |  |

Table S2. Descriptive and Bivariate analyses (χ^2^) of the responses to the individual questions relating to knowledge about rabies amongst various predictor variables in the residents of Panchkula Municipal Corporation

Continued/-

| **Variable** |  | **Family size (members)** | | **P-value** | **Children ≤ 14years** | | **P-value** |
| --- | --- | --- | --- | --- | --- | --- | --- |
|  | **N=204** | **≤5** | **≥6** |  | **Yes** | **No** |  |
| Have you heard about rabies? | |  |  | **0.44** |  |  | **0.99** |
| Yes | 195 | 139 | 56 |  | 116 | 79 |  |
| No | 9 | 8 | 1 |  | 5 | 4 |  |
| Can dogs transmit rabies? | |  |  | **0.72** |  |  | **0.31** |
| Yes | 195 | 140 | 55 |  | 114 | 81 |  |
| No | 9 | 7 | 2 |  | 7 | 2 |  |
| Can cats transmit rabies? | |  |  | **0.7** |  |  | **0.4** |
| Yes | 67 | 46 | 21 |  | 37 | 30 |  |
| No | 137 | 101 | 36 |  | 84 | 53 |  |
| Can rats transmit rabies? | |  |  | **0.74** |  |  | **0.08** |
| Yes | 67 | 47 | 20 |  | 34 | 33 |  |
| No | 137 | 100 | 37 |  | 87 | 50 |  |
| Can rabies be transmitted through animal bites? | | |  | **0.28** |  |  | ***0.04**** |
| Yes | 186 | 136 | 50 |  | 106 | 80 |  |
| No | 18 | 11 | 7 |  | 15 | 3 |  |
| Can rabies be transmitted through licks/scratches? | | |  | **0.9** |  |  | **0.05** |
| Yes | 94 | 67 | 27 |  | 49 | 45 |  |
| No | 110 | 80 | 30 |  | 72 | 38 |  |
| Is rabies fatal? | |  |  | **0.65** |  |  | **0.81** |
| Yes | 142 | 101 | 41 |  | 85 | 57 |  |
| No | 62 | 46 | 16 |  | 36 | 26 |  |
| Can rabies be prevented | |  |  | **0.99** |  |  | **0.67** |
| Yes | 162 | 117 | 45 |  | 93 | 69 |  |
| No | 42 | 30 | 12 |  | 28 | 14 |  |
| Can rabies be prevented by post -bite anti-rabies vaccines (ARV)? | | |  | **0.59** |  |  | **0.8** |
| Yes | 155 | 108 | 47 |  | 90 | 65 |  |
| No | 49 | 39 | 10 |  | 31 | 26 |  |
| Can rabies be prevented by vaccinating dogs against rabies? | | |  |  |  |  |  |
| Yes | 156 | 113 | 43 | **0.82** | 91 | 65 | **0.84** |
| No | 48 | 34 | 14 |  | 30 | 18 |  |

Continued/-

| **Variable** | **N=204** | **Social status** | | **P-value** | **Dog ownership** | | **P-value** |
| --- | --- | --- | --- | --- | --- | --- | --- |
|  |  | **Others*** | **Low** |  | **Yes** | **No** |  |
| Have you heard about rabies? | |  |  | ***0.001**** |  |  | **0.16** |
| Yes | 195 | 151 | 44 |  | 73 | 122 |  |
| No | 9 | 2 | 7 |  | 1 | 8 |  |
| Can dogs transmit rabies? |  |  |  | ***0.001**** |  |  | **0.16** |
| Yes | 195 | 150 | 45 |  | 73 | 122 |  |
| No | 9 | 3 | 6 |  | 1 | 8 |  |
| Can cats transmit rabies? |  |  |  | **0.8** |  |  | **0.75** |
| Yes | 67 | 51 | 16 |  | 23 | 44 |  |
| No | 137 | 102 | 35 |  | 51 | 86 |  |
| Can rats transmit rabies? |  |  |  | ***0.03**** |  |  | **0.75** |
| Yes | 67 | 44 | 23 |  | 23 | 44 |  |
| No | 137 | 109 | 28 |  | 51 | 86 |  |
| Can rabies be transmitted through animal bites? | | |  | **0.05** |  |  | **0.8** |
| Yes | 186 | 143 | 43 |  | 67 | 119 |  |
| No | 18 | 10 | 8 |  | 7 | 11 |  |
| Can rabies be transmitted through licks/scratches? | | |  | **0.14** |  |  | **0.3** |
| Yes | 94 | 75 | 19 |  | 38 | 56 |  |
| No | 110 | 78 | 32 |  | 36 | 74 |  |
| Is rabies fatal? |  |  |  | ***0.02**** |  |  | **0.42** |
| Yes | 142 | 113 | 29 |  | 49 | 93 |  |
| No | 62 | 40 | 22 |  | 25 | 37 |  |
| Can rabies be prevented? |  |  |  | ***0.001**** |  |  | **0.24** |
| Yes | 162 | 132 | 30 |  | 62 | 100 |  |
| No | 42 | 21 | 21 |  | 12 | 30 |  |
| Can rabies be prevented by post -bite anti-rabies vaccines (ARV)? | | |  | **0.25** |  |  | **0.34** |
| Yes | 155 | 127 | 28 |  | 59 | 96 |  |
| No | 49 | 26 | 23 |  | 15 | 34 |  |
| Can rabies be prevented by vaccinating dogs against rabies? | | |  |  |  |  |  |
| Yes | 156 | 127 | 29 | ***0.001**** | 65 | 91 | ***0.003**** |
| No | 48 | 26 | 22 |  | 9 | 39 |  |

Others*- High/middle
