# Supplementary material for: Knowledge, attitudes and practices (KAP) towards rabies and free roaming dogs (FRD) in Panchkula district of north India: A cross-sectional study of urban residents
Source: PLoS Negl Trop Dis. 2019 Apr 29;13(4):e0007384. doi: 10.1371/journal.pntd.0007384 (PMC6508743; doi:10.1371/journal.pntd.0007384)
Supplement: S3 Table — (DOCX) [file pntd.0007384.s003.docx]

Table S3. Descriptive and Bivariate analyses (χ^2^) of the responses to the individual questions related to attitudes and practices about rabies amongst various predictor variables in the residents of Panchkula Municipal Corporation

| **Variables** | **N =204** | **Gender** | | **P-value** | **Age of respondents** | | **P-value** |
| --- | --- | --- | --- | --- | --- | --- | --- |
|  |  | **Male** | **Female** |  | **≤34years** | **≥35 years** |  |
| In your opinion will application of local treatments, like chilli powder and turmeric, on animal bite wounds prevent rabies? | | | | **0.47** |  |  | **0.67** |
| Yes | 113 | 64 | 49 |  | 74 | 39 |  |
| No | 91 | 56 | 35 |  | 57 | 34 |  |
| In your opinion should animal bite wounds be washed with soap and water to reduce chances of rabies infection? | | | | **0.39** |  |  | ***0.001**** |
| Yes | 124 | 70 | 54 |  | 69 | 55 |  |
| No | 80 | 50 | 30 |  | 62 | 18 |  |
| In your opinion is it necessary to go to hospital if someone is bitten by a dog, even if the injury is not severe? | | | | **0.58** |  |  | **0.91** |
| Yes | 174 | 101 | 73 |  | 112 | 62 |  |
| No | 30 | 19 | 11 |  | 19 | 11 |  |
| Can rabies be controlled by restricting the size of the stray dog population? | | |  | **0.11** |  |  | **0.7** |
| Yes | 143 | 79 | 64 |  | 93 | 50 |  |
| No | 61 | 41 | 20 |  | 38 | 23 |  |
| If you saw a dog with signs of rabies would you inform the municipal authorities? | | |  | **0.75** |  |  | ***0.001**** |
| Yes | 113 | 63 | 50 |  | 62 | 51 |  |
| No | 91 | 57 | 34 |  | 69 | 22 |  |

Continued/-

| **Variables** | **N =204** | **Family size** | | **P-value** | **Children ≤ 14 years** | | **P-value** |
| --- | --- | --- | --- | --- | --- | --- | --- |
|  |  | **≤5** | **≥6** |  | **Yes** | **No** |  |
| In your opinion will application of local treatments, like chilli powder and turmeric, on animal bite wounds prevent rabies? | | | | **0.16** |  |  | **0.39** |
| Yes | 113 | 77 | 36 |  | 70 | 43 |  |
| No | 91 | 70 | 21 |  | 51 | 40 |  |
| In your opinion should animal bite wounds be washed with soap and water to reduce chances of rabies infection? | | | | **0.39** |  |  | **0.51** |
| Yes | 124 | 92 | 32 |  | 66 | 58 |  |
| No | 80 | 55 | 25 |  | 55 | 25 |  |
| In your opinion is it necessary to go to hospital if someone is bitten by a dog even if the injury is not severe? | | | | **0.47** |  |  | **0.62** |
| Yes | 174 | 127 | 47 |  | 102 | 72 |  |
| No | 30 | 20 | 10 |  | 19 | 11 |  |
| Can rabies be controlled by restricting the size of the stray dog population? | | | | **0.48** |  |  | **0.13** |
| Yes | 143 | 101 | 42 |  | 80 | 63 |  |
| No | 61 | 46 | 15 |  | 41 | 20 |  |
| If you saw a dog with signs of rabies would you inform the municipal authorities? | | | | **0.2** |  |  | **0.99** |
| Yes | 113 | 85 | 28 |  | 67 | 46 |  |
| No | 91 | 62 | 29 |  | 54 | 37 |  |

Continued/-

| **Variables** | **N=204** | **Social status** | | **P-value** | **Dog ownership** | | **P-value** |
| --- | --- | --- | --- | --- | --- | --- | --- |
|  |  | **Others*** | **Low** |  | **Yes** | **No** |  |
| In your opinion will application of local treatments, like chilli powder and turmeric, on animal bite wounds prevent rabies? | | | | **0.12** |  |  | **0.55** |
| Yes | 113 | 80 | 33 |  | 39 | 74 |  |
| No | 91 | 73 | 18 |  | 35 | 56 |  |
| In your opinion should animal bite wounds be washed with soap and water to reduce chances of rabies infection? | | | | **0.58** |  |  | **0.91** |
| Yes | 124 | 26 | 98 |  | 44 | 80 |  |
| No | 80 | 25 | 55 |  | 30 | 50 |  |
| In your opinion is it necessary to go to hospital if someone is bitten by a dog even if the injury is not severe? | | | | ***0.01**** |  |  | **0.38** |
| Yes | 174 | 138 | 36 |  | 61 | 113 |  |
| No | 30 | 15 | 15 |  | 13 | 17 |  |
| Can rabies be controlled by restricting the size of the stray dog population? | | | | ***0.01**** |  |  | **0.5** |
| Yes | 143 | 115 | 28 |  | 50 | 93 |  |
| No | 61 | 38 | 23 |  | 24 | 37 |  |
| If you saw a dog with signs of rabies would you inform the municipal authorities? | | | | ***0.001**** |  |  | **0.24** |
| Yes | 113 | 97 | 16 |  | 45 | 68 |  |
| No | 91 | 56 | 35 |  | 29 | 62 |  |

Others*- High/middle
