# Supplementary material for: Knowledge, attitudes and practices (KAP) towards rabies and free roaming dogs (FRD) in Panchkula district of north India: A cross-sectional study of urban residents
Source: PLoS Negl Trop Dis. 2019 Apr 29;13(4):e0007384. doi: 10.1371/journal.pntd.0007384 (PMC6508743; doi:10.1371/journal.pntd.0007384)
Supplement: S4 Table — (DOCX) [file pntd.0007384.s004.docx]

Table S4. Descriptive and Bivariate analyses (χ^2^) of the response to the individual questions related to attitudes and practices towards free-roaming dogs amongst various predictor variables in the residents of Panchkula Municipal Corporation

| **Variable** | **N=204** | **Gender** | | **P-value** | **Age of respondents** | | **P-value** |
| --- | --- | --- | --- | --- | --- | --- | --- |
|  |  | **Male** | **Female** |  | **≤34 years** | **≥35 years** |  |
| Do you feel FRD in your locality are useful to society? | | |  | **0.08** |  |  | ***0.006**** |
| Yes | 55 | 27 | 28 |  | 27 | 28 |  |
| No | 149 | 93 | 56 |  | 104 | 45 |  |
| Do you think that the FRD in your locality are a nuisance or a problem for the society? | | |  | **0.24** |  |  | ***0.02**** |
| Yes | 138 | 85 | 53 |  | 96 | 42 |  |
| No | 66 | 35 | 31 |  | 35 | 31 |  |
| Do you think that the FRD are a threat to human health? | | |  | ***0.02**** |  |  | **0.9** |
| Yes | 160 | 101 | 59 |  | 103 | 57 |  |
| No | 44 | 19 | 25 |  | 28 | 16 |  |
| Do you ever feed FRD? | | |  | **0.53** |  |  | **0.18** |
| Yes | 148 | 89 | 59 |  | 91 | 57 |  |
| No | 56 | 31 | 25 |  | 40 | 16 |  |
| If you see an injured FRD would you take it to a veterinarian? | | |  | **0.07** |  |  | ***0.002**** |
| Yes | 78 | 52 | 26 |  | 38 | 40 |  |
| No | 126 | 68 | 58 |  | 93 | 33 |  |
| In your opinion should people who feed / shelter these dogs take responsibility for their health and vaccination? | | |  | **0.9** |  |  | **0.7** |
| Yes | 111 | 64 | 47 |  | 68 | 43 |  |
| No | 93 | 57 | 36 |  | 63 | 30 |  |
| In your opinion is it the responsibility of the government to take care of health of FRD? | | |  | **0.38** |  |  | **0.37** |
| Yes | 182 | 109 | 73 |  | 115 | 67 |  |
| No | 22 | 11 | 11 |  | 16 | 6 |  |

Continued/-

| **Variable** | **N=204** | **Family size** | | **P value** | **Children ≤ 14years** | | **P-value** |
| --- | --- | --- | --- | --- | --- | --- | --- |
|  |  | **≤5** | **≥6** |  | **Yes** | **No** |  |
| Do you feel FRD in your locality are useful to society? | | |  | **0.89** |  |  | **0.56** |
| Yes | 55 | 40 | 15 |  | 27 | 28 |  |
| No | 149 | 107 | 42 |  | 94 | 55 |  |
| Do you think that the FRD in your locality are a nuisance or a problem for the society? | | |  | **0.41** |  |  | ***0.02**** |
| Yes | 138 | 97 | 41 |  | 89 | 49 |  |
| No | 66 | 50 | 16 |  | 32 | 34 |  |
| Do you think that the FRD are a threat to human health? | | |  | **0.3** |  |  | **0.28** |
| Yes | 160 | 118 | 42 |  | 98 | 62 |  |
| No | 44 | 29 | 15 |  | 23 | 21 |  |
| Do you ever feed FRD? | | |  | **0.63** |  |  | **0.12** |
| Yes | 148 | 108 | 40 |  | 83 | 65 |  |
| No | 56 | 39 | 17 |  | 38 | 18 |  |
| If you see an injured FRD would you take it to a veterinarian? | | |  | **0.22** |  |  | **0.71** |
| Yes | 78 | 60 | 18 |  | 45 | 33 |  |
| No | 126 | 87 | 39 |  | 76 | 50 |  |
| In your opinion should people who feed / shelter these dogs take responsibility for their health and vaccination? | | |  | **0.75** |  |  | **0.6** |
| Yes | 111 | 81 | 30 |  | 64 | 47 |  |
| No | 93 | 66 | 27 |  | 57 | 36 |  |
| In your opinion is it the responsibility of the government to take care of the health of the FRD? | | |  | **0.28** |  |  | **0.37** |
| Yes | 182 | 129 | 53 |  | 106 | 76 |  |
| No | 22 | 18 | 4 |  | 15 | 7 |  |

Continued/-

| **Variable** | **N =204** | **Social status** | | **P- value** | **Dog ownership** | | **P-value** |
| --- | --- | --- | --- | --- | --- | --- | --- |
|  |  | **Others*** | **Low** |  | **Yes** | **No** |  |
| Do you feel FRD in your locality are useful to society? | | |  | **0.08** |  |  | ***0.008**** |
| Yes | 55 | 46 | 9 |  | 28 | 27 |  |
| No | 149 | 107 | 42 |  | 46 | 103 |  |
| Do you think that the FRD in your locality are a nuisance or a problem for the society? | | |  | ***0.01**** |  |  | **0.11** |
| Yes | 138 | 96 | 42 |  | 45 | 93 |  |
| No | 66 | 57 | 9 |  | 29 | 37 |  |
| Do you think that the FRD are a threat to human health? | | |  | ***0.02**** |  |  | ***0.03**** |
| Yes | 160 | 114 | 46 |  | 52 | 108 |  |
| No | 44 | 39 | 5 |  | 22 | 22 |  |
| Do you ever feed FRD? | | |  | **0.15** |  |  | **0.91** |
| Yes | 148 | 107 | 41 |  | 54 | 94 |  |
| No | 56 | 46 | 10 |  | 20 | 26 |  |
| If you see an injured FRD would you take it to a veterinarian? | | |  | **0.07** |  |  | **0.81** |
| Yes | 78 | 53 | 25 |  | 26 | 52 |  |
| No | 126 | 100 | 26 |  | 48 | 78 |  |
| In your opinion should people who feed / shelter these dogs take responsibility for their health and vaccination? | | |  | **0.81** |  |  | ***0.03**** |
| Yes | 111 | 84 | 27 |  | 33 | 78 |  |
| No | 93 | 69 | 24 |  | 41 | 52 |  |
| In your opinion is it the responsibility of the government to take care of the health of FRD? | | |  | **0.79** |  |  | **0.06** |
| Yes | 182 | 137 | 45 |  | 62 | 120 |  |
| No | 22 | 16 | 6 |  | 12 | 10 |  |

Others*- High/middle
